# A *Felis catus* proteomic dataset and PeptideAtlas resource to explore the proteome of the domestic cat

**DOI:** 10.1016/j.dib.2026.113185

**Published:** 2026-08-21

**Authors:** Ulrike Kusebauch, Zhi Sun, Panga J. Reddy, Phillip Watson, David Allaway, Robert L. Moritz

**Affiliations:** a Institute for Systems Biology, 401 Terry Ave N, Seattle, WA 98109, USA; b Waltham Petcare Science Institute, Mars Petcare, Waltham-on-the-Wolds, Melton Mowbray, Leicestershire, LE14 4RT, UK

**Keywords:** Felis catus, Cat proteome, PeptideAtlas, Cell lines, Tissues, Serum, Data-dependent acquisition

## Abstract

Domestic cats (*Felis catus*) are one of the most common companion animals, and the health status and well-being of cats is becoming increasingly important. Several aspects of feline biology are vastly understudied relative to the importance of cats in society. These include robust methods to identify and measure proteins, which are critical for fundamental understanding and disease diagnosis. A searchable proteome map for cats is not available yet and here we measured immortalized feline cells, various tissues and serum using data-dependent acquisition (DDA) mass spectrometry to provide a foundational map of the cat proteome. Data were analyzed using the Trans-Proteomic Pipeline with stringent criteria and low false-discovery rate (FDR). The dataset consisting of 157 acquired files is presented as a first *Felis catus* PeptideAtlas build, a high-quality, publicly available resource of the proteome of the domestic cat that reports the identification of 242,844 unique peptides representing 8972 feline proteins or 45.6% of the annotated reference proteome. This resource can be mined for other studies and expanded as new data become available. The *Felis catus* PeptideAtlas and related data are publicly available via https://peptideatlas.org/builds/feline and ProteomeXchange (PXD075481).

## Graphical abstract

**Figure:**
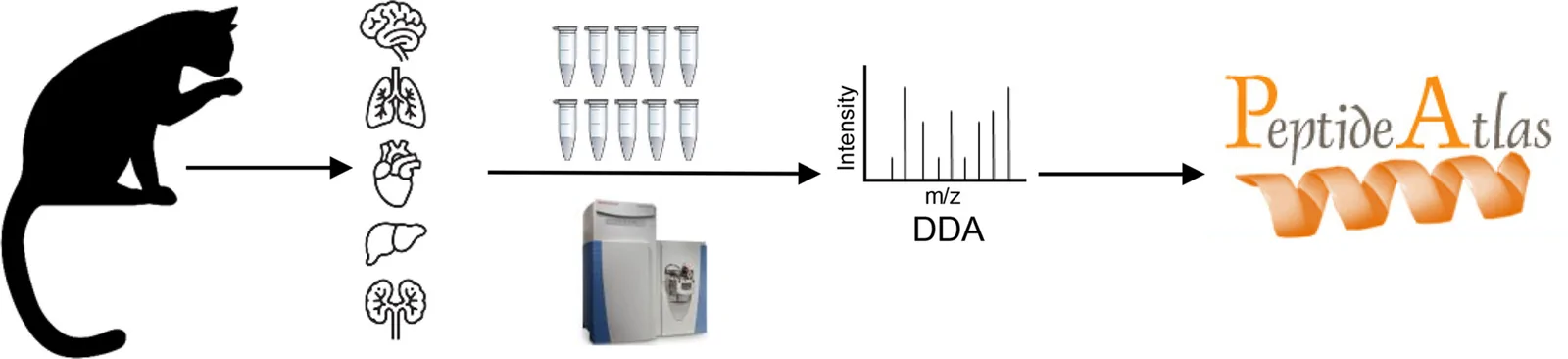

Specifications Table

**Table:** 

Subject	Biology
Specific subject area	*Felis catus* proteome; profiling of feline cells, tissues and serum; resource to study the proteome of the domestic cat
Type of data	Mass spectrometry data, Raw and Processed Data, Figures, Table, Web-resource
Data collection	*Felis catus* G355–5 and CRFK cells, feline tissue and feline serum (from post-mortem and remnant material) were reduced, alkylated and trypsin digested, followed by liquid-chromatography mass-spectrometry analysis (LC-MS/MS) in data-dependent acquisition mode (DDA) using a Q Exactive HF mass spectrometer (Thermo-Fisher Scientific) coupled to an Easy-nLC 1100 system (Thermo-Fisher Scientific). Data were processed.
Data source location	Institute for Systems Biology, Seattle, WA, USA
Data accessibility	Repository name: PRIDE Data identification number: Data identification number: PXD075481 [1] Direct URL to data: https://www.ebi.ac.uk/pride/archive/projects/PXD075481, https://peptideatlas.org/builds/feline*.*
Related research article	None

## 1 Value of the Data

• This data-dependent acquisition dataset, acquired from feline cells, tissues and serum, provides a comprehensive view of the proteome of the domestic cat (*Felis catus*).
• These mass-spectrometry data are presented through a publicly available web-portal named *Felis catus* PeptideAtlas.
• The *Felis catus* PeptideAtlas resource reports the identified peptides, proteins and mass spectra along with several attributes, serves as a reference for other studies, and facilitates the selection of optimal peptides for the development of targeted quantitative assays.
• These data support proteomics-based veterinary research to understand biological processes in the feline proteome and to develop biomarker candidates.
• These data provide a foundation of the feline proteome and the *Felis catus* PeptideAtlas can be expanded by uniformly reprocessing current and new datasets as they become available.

## 2 Background

With 49 million households owning cats in the United States alone, the well-being and aging of companion cats becomes increasingly important [2,3]. Several areas of feline biology remain largely unexplored relative to the central role cats play in everyday society, including the lack of reliable methods for protein identification and quantification. The ability to profile all proteins of an organism provides the basis to understand health and disease, biological functions, protein-protein interactions, and may lead to the development of new drugs. Monitoring changes in protein abundances enables early disease detection, tracking of progression and individualized treatment. In recent years, proteomic approaches have been applied in veterinary science with a focus on farm animals and their products, likely due to their economic importance [[4], [5], [6]]. So far, relatively few studies have investigated the feline proteome and analyses often occurred in context of a specific condition or biospecimen [7,8]. However, a comprehensive proteome map for felines, comparable to those for humans, commonly studied model organisms and recently for dogs [9], has not been established yet. We developed a foundational resource of the proteome of *Felis catus,* the domestic cat, to enable and accelerate quantitative proteomics research and to improve the well-being of feline companions.

## 3 Data Description

**Fig. 1:**
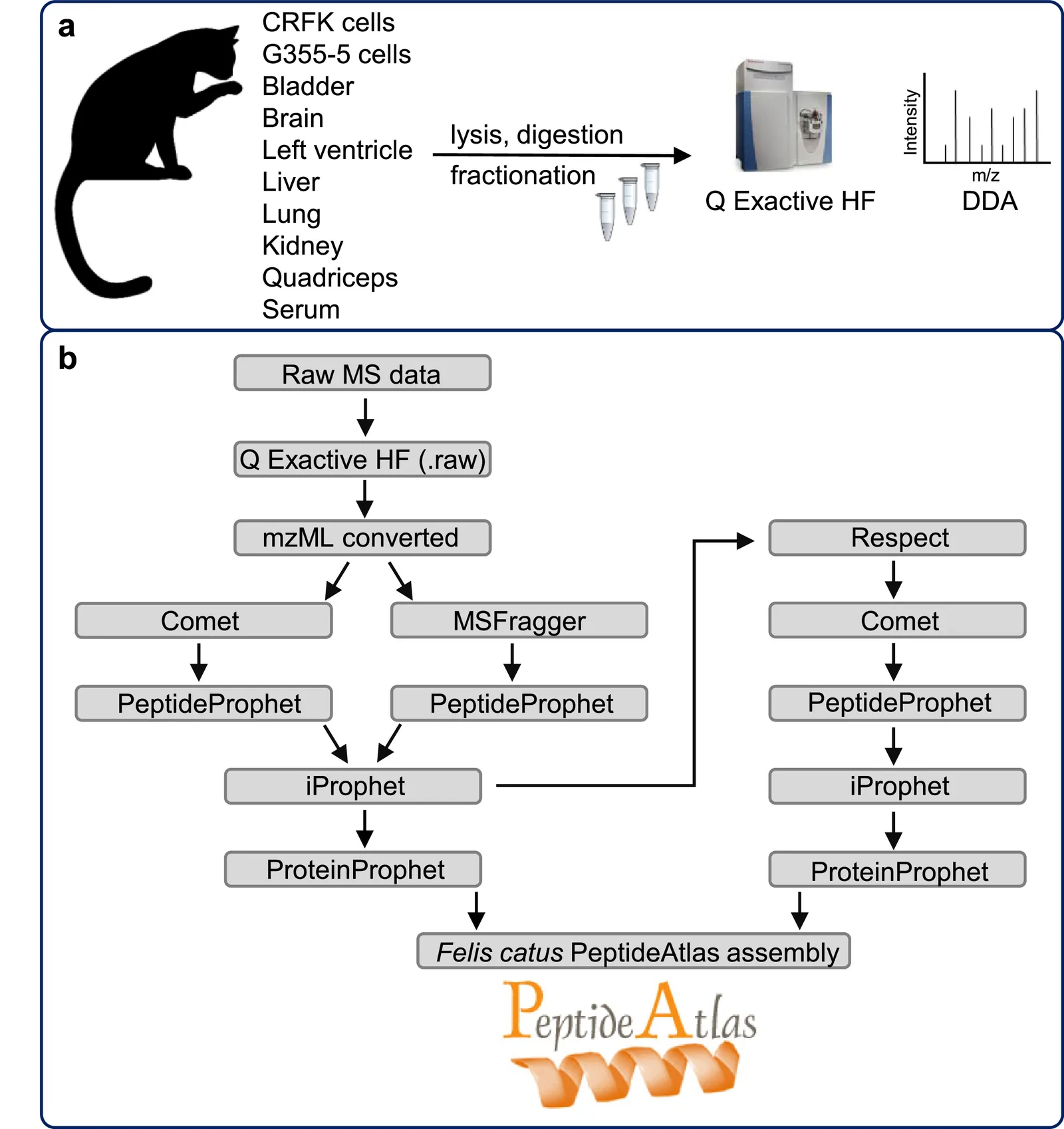
**Scheme of Feline PeptideAtlas assembly. (a)** Analysis of feline cells, tissues and serum by data-dependent (DDA) acquisition mass spectrometry. **(b)** Data analysis protocol to generate the *Felis catus* PeptideAtlas build from DDA mass spectrometry data. Q Exactive HF (.raw) data were converted and searched with Comet and MSFragger. Data were further processed with the Trans-Proteomic Pipeline including PeptideProphet, iProphet, reSpect and ProteinProphet.

To profile the proteome of the domestic cat, *Felis catus,* we cultured G355–5 astrocyte brain and CRFK kidney cells, and analyzed feline tissue including bladder, left ventricle (heart), lung, quadriceps femoris (skeletal muscle), brain, kidney, liver, as well as serum. The samples were processed by protein digestion, comprising reduction of sulfhydryl bonds and alkylation of cysteines, and were analyzed on a Q Exactive HF mass spectrometer **(**Fig. 1a**)**. Data from both cell lines were acquired in data-dependent acquisition (DDA) mode with gradients of 2 h, 4 h and 8 h length. One aliquot of each cell line was subjected to high-pH reversed-phase fractionation resulting in 32 fractions that subsequently were combined into eight fractions and analyzed by DDA with a 1 h gradient, followed by replicate analysis injecting a higher sample concentration. In addition, G355–5 cells were subjected to acetone-precipitation and samples analyzed with a 4 h gradient. Tissue and serum samples were acquired in DDA mode in three replicate runs using a 4 h gradient. In addition, kidney tissue was used as an exploratory sample and subjected to high-pH fractionation, the resulting eight fractions were acquired in DDA mode with a 2 h gradient. In total we acquired 157 runs from which we assembled a foundational proteome map and developed a *Felis catus* PeptideAtlas resource. Our goal was not to compare methods but to obtain confident peptide and protein identifications.

PeptideAtlas (www.peptideatlas.org) is a curated, multi-species compendium of peptides and their associated proteins which have been identified through discovery mass spectrometry experiments. It serves as a valuable resource for data mining, experimental planning, genome annotation refinement, and the development of targeted proteomic strategies, among other applications. The acquired mass spectrometry data were converted to mzML format and searched with Comet [10] and MSFragger [11] against the UniProtKB *Felis catus* proteome (UP000011712, cat, *Felis silvestris catus,* downloaded October 2023) containing 19,655 entries which represent one protein sequence per gene **(**Fig. 1b**)**. The search results were processed in a standardized approach with the Trans-Proteomic Pipeline (TPP) [12] including PeptideProphet [13], iProphet [14] and reSpect [15] for statistical validation of peptide spectrum matches (PSMs) and distinct peptide identifications. ProteinProphet [16] was used to infer protein identifications from confidently identified peptides (see Methods). We defined so called experiment tags by grouping the data based on tissue type, serum and cell line **(**Table 1**)**. For each experiment, stringent false discovery rate (FDR) thresholds were applied at the spectrum, peptide and protein levels, considering both model-based and decoy count-based FDR estimates, to report peptide and protein identifications.

**Table 1:** **Data contribution in the Feline PeptideAtlas from cells, tissues and serum.** The number of MS runs, identified spectra above the confidence threshold, matching distinct and unique peptides contributing to each experiment are summarized together with the number of cumulative peptides, corresponding distinct canonical and unique canonical proteins. In addition, the number of unique all proteins (not limited to canonical proteins), added canonical proteins (order-dependent increase in the number of proteins gaining canonical status by the addition of this experiment), and cumulative canonical proteins (order-dependent increase) are provided.

**Experiment Tag**	**MS Runs**	**Identified** **Spectra**	**Distinct Peptides**	**Unique Peptides**	**Cumulative Peptides**	**Distinct Canonical Proteins**	**Unique Canonical Proteins**	**Unique All Proteins**	**Added Canonical Proteins**	**Cumulative Canonical Proteins**
CRFK_2hrs	5	351,460	24,936	325	24,935	2618	0	3	2618	2618
CRFK_4hrs	9	1187,766	33,068	995	35,074	3195	0	29	683	3301
CRFK_8hrs	9	1964,420	34,953	1298	41,070	3408	1	51	390	3691
CRFK_Fractionation	16	266,469	36,009	4039	51,136	3802	35	254	671	4362
G355–5_2hrs	7	143,559	17,755	421	54,983	1957	0	11	154	4516
G355–5_4hrs	18	1524,849	42,381	3984	65,512	3462	2	69	313	4829
G355–5_8hrs	9	1943,267	37,547	1786	68,523	3413	1	51	123	4952
G355–5_Fractionation	24	282,651	42,938	7496	77,708	3865	32	237	301	5253
Bladder	9	437,547	12,814	1736	82,690	951	5	24	154	5407
Left_ventricle	5	458,831	20,358	3591	91,560	1303	25	55	179	5586
Lung	6	252,702	15,988	1356	95,418	1421	10	50	196	5782
Quadriceps	6	507,451	13,126	3384	99,014	697	23	41	46	5828
Brain	7	503,848	30,220	10,195	110,140	2522	262	486	493	6321
Kidney	20	889,764	31,080	7509	118,716	2472	94	199	219	6540
Liver	6	401,248	18,867	2632	121,365	1627	16	48	51	6591
Serum	1	15,027	2514	1711	123,076	111	15	22	26	6617

**Fig. 2:**
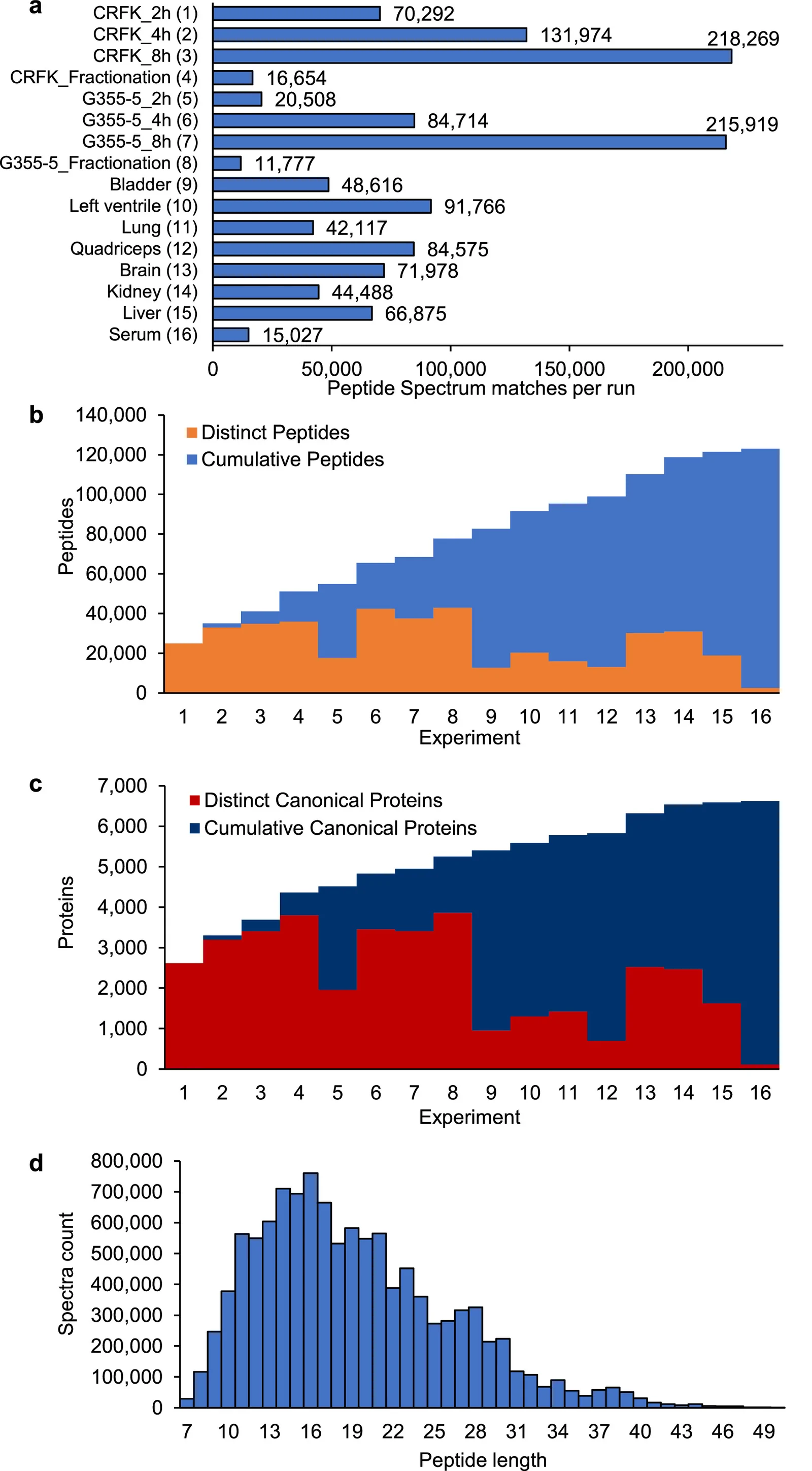
**Feline PeptideAtlas overview. (a)** The graph depicts the peptide spectrum matches per mass spectrometry run for each experiment category. Experiments were defined based on sample type with experiment 1–8 denoting cell line samples and different mass spectrometry analysis times and with experiment 9–16 denoting different feline tissue and serum sample analyses. The experiment group is indicated in parenthesis. **(b)** The graph depicts the number of identified distinct peptides in each experiment (orange) and their cumulative count (blue) that contributed to the Feline PeptideAtlas build. Experiment categories are as described above. **(c)** The graph shows the number of distinct canonical proteins in each experiment (red) and the cumulative number of proteins in each experiment (blue) inferred from the identified peptides. Experiment categories are as described above. **d)** Peptide length distribution of observed distinct peptides in the *Felis catus* PeptideAtlas build.

All data are publicly available through the PRIDE data repository [17] with dataset identifier PXD075481. The data structure in PRIDE mirrors the 16 defined experiments in Table 1 and Fig. 2. In more detail, we grouped the respective data by cell line, tissue type and serum, further subdividing the cell line mass spectrometry (MS) runs by gradient length and fractionated samples. We then provided for each experiment tag separate folders with the corresponding data types including raw data (.raw), converted data (.mzML), Comet search results (pep.xml), MSFragger search results (pep.xml), the TPP processed iProphet results of the Comet and MSFragger search (ipro.pep.xml), the reSpect derived data (.mzML, two rounds of reSpect) and the iProphet results (ipro.pep.xml, of two rounds of respect) resulting in 110 shared data folders (16 experiment tags with their corresponding seven data types, no reSpect results for serum) including a total 972 data files. In addition, we share the used search database as part of the PRIDE data submission and provide a detailed overview (overview_feline_data_final.xlsx) of the uploaded data files used to develop the *Felis catus* PeptideAtlas resource. The overview in Excel format lists the file name, the number of files in each folder, the file type, sample type and provides a brief description as additional help.

The acquired data comprises 11.1 million identified spectra (0.0001 FDR) used to assemble the *Felis catus* PeptideAtlas. These 11.1 million spectra map to 242,844 modified peptides denoting 123,076 distinct stripped peptides (0.0014 FDR) which represent 8972 feline proteins (0.015 FDR) or 45.6% of the predicted UniProtKB *Felis catus* proteome based on 19,655 unique genes and their representative protein sequences. The identified 8972 proteins in the *Felis catus* PeptideAtlas were then assigned to confidence categories as described in van Wijk et al. [18]. We report 6617 canonical feline proteins, these are identifications at the highest confidence level (0.0001 FDR). Non-detected proteins are classified at the lowest confidence level, and intermediate levels are described using a tiered system [18]. These include 67 indistinguishable representatives (protein identifications based on peptides that map to several proteins), 19 proteins with insufficient evidence (based on the identification of peptides with less than nine amino acids), 252 marginally distinguished proteins (proteins that share several peptides with a canonical protein but also have one unique peptide with less than nine amino acids), and a larger group of 1336 proteins classified as weak (proteins with one unique peptide with a minimum of nine amino acids and potentially other shorter or shared peptides but lack a second non-nesting, unique peptide with nine amino acids), totaling 8972 proteins reported in the *Felis catus* PeptideAtlas.

A graphical overview of the *Felis catus* PeptideAtlas build is provided in Fig. 2. The identified peptide spectrum matches per mass spectrometry run for each experiment category are depicted in Fig. 2a. Experiments were defined based on the sample type with experiment 1–8 denoting cell line samples and different mass spectrometry analysis times and with experiment 9–16 denoting the different feline tissue and serum sample analyses. Fig. 2b displays the number of identified distinct peptides in each experiment and their cumulative count and Fig. 2c shows the number of canonical proteins and their cumulative count that contributed to the *Felis catus* PeptideAtlas build. Lastly, Fig. 2d depicts the peptide length distribution of the identified distinct peptides in the *Felis catus* PeptideAtlas build.

We established here a foundational resource of the feline proteome that covers 45.6% of the annotated reference proteome applying stringent analysis criteria. This resource is publicly available and can be expanded by including other high-quality proteomic datasets of feline samples and by uniformly reprocessing all data to provide a next generation *Felis catus* PeptideAtlas build with increased proteome coverage. As feline biology is grossly understudied compared to many other mammalian species, this foundational resource will enable and accelerate quantitative proteomics research unlocking a deeper understanding of health and disease, the discovery of biomarkers for early disease detection and the identification of novel treatment paths in support of feline health and wellbeing.

## 4 Experimental Design, Materials and Methods

### 4.1 Cell culture and sample preparation

Felis catus G355–5 astrocyte brain cells (ATCC Catalog No CRL-2033) were cultured in McCoy's 5A (Modified) Medium (ATCC Catalog No 30–2007) with heat-inactivated fetal bovine serum added at a final concentration of 10%. Felis catus CRFK kidney cells (ATCC Catalog No CCL-94) were cultured in Eagle's Minimum Essential Medium (ATCC Catalog No 30–2003) with horse serum (Gibco Catalog No 16,050,122) at a final concentration of 10%. Cells were tested for mycoplasma contamination using the Universal Mycoplasma Detection Kit (ATCC Catalog No 30–1012 K), mycoplasma was not detected. Cells were harvested and pelleted (1000 rpm, 15 min, 4 °C). Cells pellets were washed with phosphate-buffered saline (PBS, pH 7.4) and stored at −80 °C for later use. Frozen cell pellets were resuspended in 8 M urea, 100 mM ammonium bicarbonate (Thermo-Fisher Scientific Catalog No AC393210010) with cOmplete protease inhibitor cocktail (Roche, SigmaAldrich Catalog No 04,693,116,001). Cells were lysed by sonication (2 s pulse, 2 s gap, 10 cycles at 25% amplitude) followed by lysate centrifugation (25,000 rpm, 30 min, 4 °C). The soluble proteome supernatant was collected and quantified using the Protein Assay Kit II (Bio-Rad Catalog No 5000,002). 100 µg total protein from each cell line were reduced with 5 mM Tris (2-carboxyethyl) phosphine hydrochloride (TCEP, Thermo-Fisher Scientific Catalog No PG82080, 30 min, 80 °C), alkylated with 10 mM iodoacetamide (IAM, Thermo-Fisher Scientific Catalog No 122,271,000, 30 min, room temperature, darkness), and diluted with 50 mM ammonium bicarbonate to a urea concentration of 1 M. Samples were digested with Trypsin Gold (Promega Catalog No V5280) at a ratio of 1:30 trypsin to protein for 16 h at 37 °C. The digestion was stopped by adding 0.1% Trifluoroacetic acid (TFA, Honeywell Catalog No 6001,761). Peptides were cleaned-up using C-18 Spin Columns (Thermo-Fisher Scientific Catalog No 89,934) per manufacturer’s instruction and dried under centrifugal evaporation (Savant, Thermo-Fisher Scientific). An aliquot of each cell line sample was subjected to high-pH reversed phase peptide fractionation and 32 fractions were subsequently combined into eight fractions for liquid-chromatography mass spectrometry (LC-MS) analysis. In addition, G355–5 cells were lysed and subjected to acetone-precipitation. Briefly, after cell lysis as described above, the soluble proteome supernatant was cooled on ice. Six volumes of cold acetone (cooled to −20 °C) were added, mixed, and incubated for 16 h at −20 °C. The sample was centrifuged (25,000 rpm, 15 min, 4 °C) to pellet proteins. The supernatant was discarded and the remaining acetone allowed to evaporate. The pellet was dissolved in 50 mM ammonium bicarbonate and subjected to proteolytic digestion as described above.

### 4.2 Feline tissue and serum material

Feline tissue was sourced from a healthy one year old neutered male cat, euthanized by a veterinarian following a road traffic accident. Samples included bladder, left ventricle (heart), lung, quadriceps femoris (skeletal muscle), brain, kidney and liver. A serum sample from a healthy cat was provided by the Waltham Petcare Science Institute. Excised tissues and serum were stored at −80⁰C.

### 4.3 Tissue sample preparation

Feline tissues were thawed and rinsed five times with PBS (pH 7.4). 150 mg of each tissue was homogenized in 500 µl 8 M urea, 50 mM ammonium bicarbonate buffer with cOmplete protease inhibitor cocktail (Roche) using a PreCellys Evolution (Bertin Technologies). Protein content of each sample was determined using the Protein Assay Kit II (Bio-Rad). An aliquot of 200 µg protein from each tissue was reduced with 5 mM TCEP (15 min, 55 °C), alkylated with 15 mM IAM (30 min, room temperature, darkness) with shaking at 800 rpm on a Thermomixer C (Eppendorf), and diluted with 50 mM ammonium bicarbonate to a urea concentration of 1 M. Samples were digested with Trypsin Gold (Promega) at a ratio of 1:30 trypsin to protein for 16 h at 37 °C. The digestion was stopped by adding 0.1% TFA and peptides cleaned by using C-18 Spin Columns per manufacturer’s instruction. Samples were dried under Savant centrifugal evaporation.

### 4.4 Serum sample preparation

Feline serum was thawed on ice, 100 µL serum were centrifuged (4000 x g, 10 min), and the pellet of tissue debris discarded. The protein content of the serum sample was determined by micro bicinchoninic acid assay (BCA, Thermo-Fisher Scientific Catalog No 23,235). Serum was subjected to depletion of the 14 most abundant feline orthologue proteins using the human multiple affinity removal system (MARS Hu-14 Catalog No 5188–6558, 4.6 × 100 mm, Agilent Technologies) by HPLC according to the manufacturer’s protocol. The depleted fraction was collected in 1.25 mL of MARS Buffer A (Agilent Catalog No 5185–5987). The column was regenerated by MARS Buffer B (Agilent Catalog No 5185–5988) before re-equilibration in MARS Buffer A. The depleted sample was denatured by adding 600 mg urea to 8 M final concentration, reduced with 5 mM dithiothreitol (30 min, 55 °C) and alkylated with 14 mM IAM (30 min, room temperature, darkness). The sample was desalted using a HiPrep 26/10 desalting column (Cytiva Catalog No 17,508,701) with a 1100 HPLC system (Agilent Technologies), and automatically collected in 2 mL of 1% ammonium bicarbonate. The protein concentration of the desalted samples was determined by BCA and samples were digested with 4 µg of Trypsin Gold for 16 h at 37 °C. The digested sample was acidified with 5 µL 1% formic acid (FA, Thermo-Fisher Scientific Honeywell Catalog No, 6000,616) and dried under centrifugal vacuum evaporation.

### 4.5 Data dependent acquisition (DDA)

Peptides from each sample were reconstituted in 0.1% FA and analyzed with a high-resolution Q Exactive HF mass spectrometer (Thermo-Fisher Scientific) coupled to an Easy-nLC 1000 system (Thermo-Fisher Scientific). Peptides were loaded on an Acclaim PepMap 100 trap (2 cm, 75 µm ID, C18 3 µm, Thermo-Fisher Scientific Catalog No 164,946) and washed with 0.1% FA in water for 10 min. Peptides were separated on an analytical column (PicoChip, 105 mm, 1.9 um, REPROSIL Pur C-18-AQ, 120 Å, New Objective Catalog No NEW11080–1101) using 0.1% FA in water (A) and 0.1% FA in acetonitrile (B) (v/v) Thermo-Fisher Scientific Catalog No 325,730,025) with a flow rate of 300 nL/min. A linear gradient from 5% to 35% B in 120 min, followed by 80% B for 5 min and equilibration of the column for 10 min was applied. Further, a linear gradient from 5% to 35% B was applied in 240 min and 480 min followed by equilibration. Survey full-scan MS spectra were acquired in DDA mode in the mass range 375–1375 *m/z* at 30,000 resolution, automatic gain control (AGC) target set to 1e6 with a 50 ms maximum ion injection time (IT). Peptides were fragmented above a threshold of 2e4 by collision induced dissociation (CID) at a resolution of 15,000, AGC target 1e5, maximum IT 50ms, TopN of 40, an isolation width of 1.2 *m/z*, and a normalized collision energy of 28%. Charge state z = 1, unassigned charges and z ≥ 6 were rejected; dynamic exclusion was set to 60 s. A spray voltage of 1500 V in positive mode and an RF lens at 65% were used.

### 4.6 DDA data analysis and peptideatlas assembly

Q Exactive HF mass spectrometry raw files were converted to mzML format using ProteoWizard msConvert (version 3.0.10505) [19,20]. MS/MS spectra were associated with peptide sequences using Comet (version 2023.01 rev. 2) [10] and MSFragger (version 3.7) [11] as search engines. The protein search space included (i) the UniProtKB *Felis catus* proteome (UP000011712, cat, *Felis silvestris catus,* downloaded October 2023) containing 19,655 entries which represent one protein sequence per gene, (ii) 499 contaminant protein sequences frequently observed in proteome samples (e.g., keratins, trypsin, BSA), and (iii) a sequence-shuffled decoy counterpart. Searched peptides were allowed to be semi-tryptic with up to two missed cleavages. The search parameters included a fixed modification of +57.021464 for carbamidomethylated cysteine and variable modifications of +15.9949 for oxidized methionine and tryptophan, +42.0106 for protein n-terminal acetylation, −17.02650 for pyroglumatic acid formation from glutamine and for cyclization of n-terminal S-carbamoylmethyl-cysteine, −18.01060 for pyroglumatic acid formation from glutamic acid and +0.984016 deamidation of asparagine. A monoisotopic precursor mass error tolerance of 20 ppm was used in Comet [10] and MSFragger [11] with the isotope error setting activated. In total 157 data files were searched (Table 1).

The search results were processed and statistically validated with the open-source proteomics data analysis package Trans-Proteomic Pipeline (TPP, version v6.4.0 Incus, build 202,310,260,143–9045) [12] including PeptideProphet [13] and iProphet [14] (Fig. 1). Peptide spectrum matches (PSM) generated by each search engine were analyzed with PeptideProphet to assign each PSM a probability of being correct. PeptideProphet was run using accurate mass, variable modification count and semi-parametric models. The expectation values were used as the only contributor to the f-value for modeling. The ppm mass errors instead of Daltons were used for mass modeling. Decoy hits were reported with a probability based on the model estimates. PeptideProphet results were further processed with iProphet to refine the PSM-level probabilities and compute peptide-level probabilities based on corroborating information from the ensemble of identifications, and to combine the results from the two search engines. All data except serum were further processed using two rounds of reSpect [15] within the TPP. For the first round of reSpect, the minimum probability was set to 0 and for the second round the minimum probability was set to 0.5. The new set of .mzML files generated by both rounds of reSpect were searched using Comet (version 2023.01 rev. 2) with precursor mass tolerance set to 3.0 and isotope_error off, and processed using PeptideProphet as described above. iProphet was used to process the PeptideProphet validated reSpect results. ProteinProphet [16] was run after iProphet. To assemble a *Felis catus* PeptideAtlas build from the different experiments, each experiment was thresholded at an iProphet probability that yields a model-based PSM FDR of 0.0001. Decoy identifications were retained and used to compute final decoy-based FDRs. Contaminant protein and peptide identifications were included in the FDR calculation.

## Limitations

Our dataset provides a foundation of the *Felis catus* proteome based on the samples available to date and the technologies. Tissue samples are obtained from a single cat. This represents a single biological replicate and this dataset does not provide an overview of the natural variabilities between individuals (by age, sex, health status or breed). Access to other tissues and body fluids, alternative methods to acquire the proteome and advanced bioinformatic tools would enhance the number of peptide and protein identifications further. In addition, we considered primary modifications that occur during sample handling but not various other post-translational modifications that often require larger sample amounts and specific enrichment techniques. However, these can be included in the *Felis catus* resource once more data become available.

## Ethics Statement

Tissues were derived post-mortem from a feline euthanized by a veterinarian (Blue Pearl Pet Hospital, Tampa, FL) following a road traffic accident, with owner consent for tissue to be used for research. A serum sample from a healthy cat was provided by the Waltham Petcare Science Institute, with housing and care consistent with United Kingdom Home Office regulations. This study was approved by the WALTHAM Animal Welfare and Ethical Review Body (Ref 03,112,014) and the blood sample collection was performed under authority of the United Kingdom Regulation of Animals (Scientific Procedures) Act of 1986.

## CRediT Author Statement

**Ulrike Kusebauch:** Conceptualization, Writing – Original draft. **Zhi Sun:** Formal analysis, Writing – review and editing. **Panga J. Reddy:** Investigation, Writing – review and editing. **Phillip Watson**: Conceptualization, Writing – review and editing, Funding acquisition. **David Allaway:** Conceptualization, Resources, Writing – review and editing, Funding acquisition. **Robert L. Moritz:** Conceptualization, Resources, Writing – review and editing, Supervision, Funding acquisition.

## Data Availability

PeptideAtlasFelis catus PeptideAtlas (Original data). PRIDEDDA data of Felis Catus immortalized cells, tissues and plasma (Original data).
